# Discovery of selective inhibitors of Glutaminase-2, which inhibit mTORC1, activate autophagy and inhibit proliferation in cancer cells

**DOI:** 10.18632/oncotarget.2173

**Published:** 2014-07-08

**Authors:** Yue-Zhi Lee, Cheng-Wei Yang, Hsin-Yu Chang, Hsing-Yu Hsu, Ih-Shen Chen, Hsun-Shuo Chang, Chih-Hao Lee, Jinq-chyi Lee, Chidambaram Ramesh Kumar, Ya-Qi Qiu, Yu-Sheng Chao, Shiow-Ju Lee

**Affiliations:** ^1^ Institute of Biotechnology and Pharmaceutical Research, National Health Research Institutes, Miaoli, Taiwan; ^2^ School of Pharmacy, College of Pharmacy, Kaohsiung Medical University, Kaohsiung, Taiwan; ^3^ Department of Genetics and Complex Diseases, Division of Biological Sciences, Harvard School of Public Health, Boston, Massachusetts, USA; ^4^ Graduate Program of Biotechnology in Medicine, Institute of Molecular & Cellular Biology, National Tsing Hua University, Hsinchu, Taiwan

**Keywords:** AMPK, Autophagy, Glutaminase, mTOR, Raptor, ULK1

## Abstract

Glutaminase, which converts glutamine to glutamate, is involved in Warburg effect in cancer cells. Two human glutaminase genes have been identified, *GLS* (*GLS1*) and *GLS2*. Two alternative transcripts arise from each glutaminase gene: first, the kidney isoform (KGA) and glutaminase C (GAC) for *GLS*; and, second, the liver isoform (LGA) and glutaminase B (GAB) for *GLS2*. While GLS1 is considered as a cancer therapeutic target, the potential role of GLS2 in cancer remains unclear. Here, we discovered a series of alkyl benzoquinones that preferentially inhibit glutaminase B isoform (GAB, GLS2) rather than the kidney isoform of glutaminase (KGA, GLS1). We identified amino acid residues in an allosteric binding pocket responsible for the selectivity. Treatment with the alkyl benzoquinones decreased intracellular glutaminase activity and glutamate levels. GLS2 inhibition by either alkyl benzoquinones or GLS2 siRNA reduced carcinoma cell proliferation and anchorage-independent colony formation, and induced autophagy via AMPK mediated mTORC1 inhibition. Our findings demonstrate amino acid sequences for selective inhibition of glutaminase isozymes and validate GLS2 as a potential anti-cancer target.

## INTRODUCTION

Cancer cells tend to exhibit the Warburg effect, in which cellular metabolisms proceed via: 1) an altered glucose metabolism characterized by a high rate of glycolysis followed by lactic acid fermentation; and 2) up-regulated glutaminolysis by increasing glutaminase activity. Glutamine is essential for cancer cell proliferation, especially in the context of tricarboxylic acid (TCA) cycle anaplerosis. Glutaminase plays an essential role converting glutamine to glutamate, which is converted by glutamate dehydrogenase into α-ketoglutarate. This α-ketoglutarate enters the TCA cycle where it is involved in the production of ATP, nucleotides, certain amino acids, lipids, and glutathione in mitochondria [1]. Recently, glutaminase has emerged as a drug target for the development of glutaminase inhibitors for the treatment of cancerous disease [2]. However, only a few series of glutaminase inhibitors for GLS1 have been identified, and only partial *in vivo* anti-cancer efficacy has been demonstrated [3-6].

There are two human glutaminase genes, *GLS* (*GLS1*) and *GLS2*, located in chromosomes 2 and 12 respectively. Two alternative transcripts arise from each glutaminase gene: the kidney isoform (KGA) and glutaminase C (GAC) for *GLS1*; and the liver isoform (LGA) and glutaminase B (GAB) for *GLS2* [7].

The dimer-to-tetramer transition is essential for the enzymatic activation of glutaminase, which is active as a tetramer. Phosphate enhances this activation by facilitating the dimer-to-tetramer transition and substrate entry to the binding pocket by competing with the product glutamate [3, 8]. Each glutaminase isoform has distinct kinetic and molecular characteristics, and the activity of each is also modulated by a variety of compounds, such as glutamate, citrate, calcium, certain long chain fatty acids, fatty acyl-CoA derivatives, TCA cycle intermediates, and protons [9], all of which are present under physiological conditions. Therefore, the kinetic and allosteric properties of the enzyme and the physiological environment are essential for regulating the enzyme activity in situ. Also, the differentiated amino acid residues or domains of the glutaminase isozymes provide potential binding pockets for selective allosteric modulators. For example, BPTES (bis-2-(5-phenylacetimido-1,2,4-thiadiazol-2-yl) ethyl sulfide), a GLS1 selective inhibitor, binds GAC through a differentiated gating loop close to the glutamine substrate binding site and locks the GAC tetramer into a nonproductive conformation [3].

In contrast to the disease indications of the KGA and GAC (GLS1) inhibitors described for anti-cancer [5, 6, 10], the biological role of GLS2 is still under exploration. GLS2 was found to regulate energy metabolism and antioxidant function as a tumor suppressor gene [11-13]when ectopically overexpressed [11]; enrichment with LGA inhibits glioma cell growth and facilitates chemotherapeutic intervention [14]. However, knock-down of GLS2 expression sensitizes cervical cancer to ionizing radiation and thus reduces tumor size through decreasing cellular glutathione and NADH [15]. Therefore, more work is needed to clarify and demonstrate the role of GLS2 in cancer cell growth.

Glutaminase inhibition blocks the conversion of glutamine to glutamate and thus disables the conversion of glutamate into α-ketoglutarate by glutamate dehydrogenase, which normally enters the TCA cycle to provide energy and bio-precursors for cancer cell growth. Autophagy is a catabolic process generally activated by such conditions of nutrient deprivation, and results in the autophagosomic-lysosomal degradation of bulk cytoplasmic contents. Autophagy is initiated and promoted by AMPK_ULK1 axis but inhibited by mTORC1 [16]. AMPK senses energy changes in cells and is activated when nutrients are depleted [17]. Rapamycin inhibits the Warburg effect [18, 19] and glutaminolysis feeds mTORC1 [20] so that a complex feedback exists between mTOR and glutaminase and Warburg effect. mTORC1 is also a critical regulator of autophagy induction and activation of mTORC1 suppresses autophagy. AMPK interacts with, phosphorylates, and activates ULK1 protein kinase, a key initiator of the autophagic process. Residue Ser317 of ULK1 is the main phosphorylation site for activation by AMPK [16]. In mammals, AMPK regulates autophagy, also involving inactivation of the mTORC1 pathway upon nutrient deficiency through two distinct pathways: phosphorylation for activation of Tuberin exchange factor or Raptor [21]. Conversely, mTOR phosphorylates ULK1 at Ser757 and disrupts the interaction between ULK1 and AMPK to inhibit autophagy [16]. In addition, the Beclin1 network also can induce and regulate autophagy via the formation of Beclin1-Vps34-Vps15 core complexes, and phagophore nucleation. The interaction of BCL2 with Beclin1 reduces autophagy. Phosphorylation of BCL2 by c-Jun N-terminal kinase 1 (JNK1) liberates Beclin1, allowing it to enter the nucleation process for autophagy [22]. Phosphorylation of Beclin1 by ULK1 at Ser15 (Ser14 in mice) is also required for full autophagic induction in mammals [23].

Natural products are a major source of inspiration in drug discovery [24, 25], and constitute a good resource for those seeking novel glutaminase inhibitors. While GLS1 is emerging as a therapeutic target for anticancer drugs [5, 6, 10], the biological role of GLS2 is still under exploration. Herein, we disclose a series of natural alkyl benzoquinones with a glutaminase inhibitory effect. Through homologous modeling and mutagenesis, the alkyl benzoquinone binding site was simulated and demonstrated to be an allosteric pocket. From the allosteric pocket, two divergently differentiated residues were found to account for the selective inhibition for GAB (an isoform of GLS2) over KGA (an isoform of GLS1). Furthermore, inhibition of glutaminase activity by the active alkyl benzoquinone AV-1 in carcinoma cells led to autophagy via AMPK-mediated ULK1 activation and mTORC1 inhibition, ultimately leading to inhibition of cancer cell growth.

## RESULTS

### Purification of the recombinant human KGA and GAB for screening glutaminase inhibitors and analysis of structure-activity relations and inhibition modes

**Figure 1:**
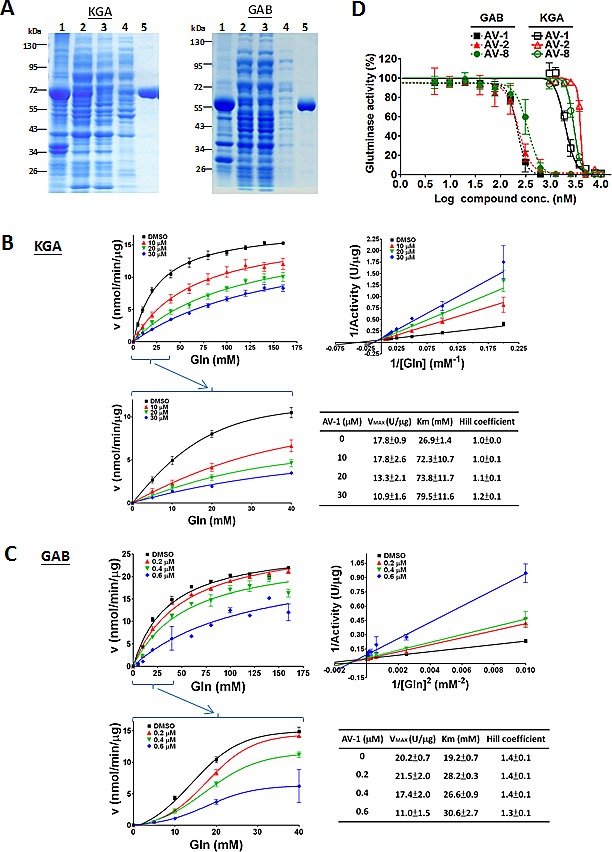
Kinetic studies on the inhibition of alkyl benzoquinones in glutaminases (A) *Escherichia coli* BL21(DE3)pLysS harboring pET28a(+)-KGA or pET28a(+)-GAB was propagated in Luria-Bertani broth medium in the presence of kanamycin, from which KGA or GAB protein expression was induced by 0.5 mM IPTG. The resultant cell lysate was sonicated and centrifugated for fractions of cell pellet (lane 1) and supernatant (lane 2). The supernatant was passed through an affinity column (HisTrapTM HP) and the flow-through was shown in lane 3, as well as the washes with 100 mM and 150 mM of imidazole for KGA(left panel) or 50 mM of imidazole for GAB(right panel) were shown in lane 4. Lane 5 shown was the eluent with 500 mM of imidazole for KGA(left panel) or 200 mM of imidazole for GAB(right panel). Molecular markers were indicated as kDa. (B) Inhibition mode of active alkyl benzoquinone AV-1 for KGA in the absence of phosphate. (C) Inhibition mode of active alkyl benzoquinone AV-1 for GAB in the absence of phosphate. The Cheng-Prusoff equation, Ki=IC_50_/(1+[S]/K_m_), was used to calculate Ki values of AV-1 for KGA=1.20±0.06μM and GAB=0.14±0.01μM. (D) Dose dependent inhibition of alkyl benzoquinones against the purified recombinant KGA and GAB in the absence of phosphate.

A collection of ~200 natural products isolated from a variety of indigenous Taiwanese plants were submitted for screening against KGA. Human recombinant KGA and GAB (Figure 1A) from *E. coli* were cloned, expressed and purified. Characterization of its enzymatic properties showed that the recombinant GAB but not KGA responded to its substrate glutamine in allosteric and positive cooperative manners (Figure 1B and 1C), as previously reported for these enzymes purified from human biopsies [26]. We first screened the compound collection described above for inhibitory activity against the recombinant KGA, and then determined the IC_50_ values against KGA and GAB for the potent compounds (Table 1, Figure 1D).

Among the active hit compounds, a series of alkyl benzoquinones from *Ardisia. virens* (Table 1, AV series) were found to exhibit inhibition of human KGA and GAB (Table 1 & Figure 1D). The keto (hydroxyl) groups at positions 1 and 4 on the benzoquinone core and the acetate group at position 2' were all found to be essential for potency for either KGA or GAB inhibition, by comparing the most potent compounds AV-1, AV-2, and AV-8 (KGA, IC_50_ values 2.1±0.1 μM; 3.9±0.4 μM; 2.9±0.1 μM; GAB, IC_50_ values 0.28±0.02 μM; 0.29±0.05μM; 0.26±0.05 μM respectively) to the other AV compounds (KGA, IC_50_ values > 40 μM; GAB, IC_50_ values > 4.7 μM) (Table 1).

**Table 1:** Natural alkyl benzoquinones inhibit the enzymatic activities of human KGA and GAB See Materials and Methods for the measurement of the IC_50_ values. Shown structures are alkyl benzoquinones and alkyl phenols isolated from plants *Ardisia virens* (AV series) and *A. kusukuensis* (AK series)[27, 28] 
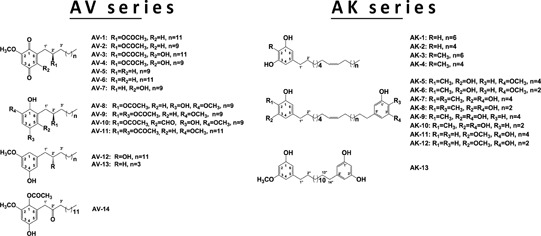

	Glutaminase	
	KGA	GAB
Compound ID	IC50 (μM)	IC50 (μM)
AV-1	2.1 ± 0.1	0.28 ± 0.02
AV-2	3.9 ± 0.4	0.29 ± 0.05
AV-3	<50	25.1 ± 4.5
AV-4	<50	<50
AV-5	<50	32.5 ± 2.7
AV-6	<50	<50
AV-7	<50	<50
AV-8	2.9 ± 0.1	0.26 ± 0.05
AV-9	<50	31.7 ± 6.7
AV-10	<50	23.1 ± 4.9
AV-11	69.0 ± 3.1	5.7 ± 1.1
AV-12	52.4 ± 15.5	7.6 ± 2.0
AV-13	52.7 ± 2.7	<50
AV-14	41.0 ± 5.2	4.7 ± 1.3
AK-1	73.0 ± 3.0	2.9 ± 0.6
AK-2	76.5 ± 0.9	2.8 ± 0.3
AK-3	76.9 ± 0.8	2.7 ± 10.4
AK-4	59.8 ± 4.6	<50
AK-5	70.0 ± 1.6	2.3 ± 0.4
AK-6	36.7 ± 3.2	2.5 ± 0.3
AK-7	17.5 ± 2.6	2.9 ± 0.8
AK-8	13.9 ± 1.5	3.1 ± 0.5
AK-9	11.9 ± 1.2	2.3 ± 0.5
AK-10	11.7 ± 1.3	2.1±0.1
AK-11	13.4 ± 2.2	2.9±0.5
AK-12	9.6 ± 1.2	2.9 ± 0.6
AK-13	14.4 ± 2.3	2.8 ± 0.4
DON	59.9 ± 8.8	ND*
BPTES	0.08 ± 0.01	63.6 ±3.8

*ND: not determined

As the AV compounds were also found to be selective for GAB over KGA, the inhibition mode of AV-1 (the most active) was investigated. AV-1 inhibited both KGA and GAB in a mixed non-competitive inhibition; when AV-1 concentration was increased from 0 to 30 μM, the K_m_ values increased by 2.9- and 1.6-fold and the V_max_ values decreased to 61% and 54% for KGA and GAB respectively (Figure 1 B & 1C). AV-1 exhibited Ki values of 1.20±0.06 μM for KGA and 0.14±0.01 μM for GAB, as calculated using the Cheng-Prusoff equation; the AV-1 exerted a positive cooperative manner with the substrate glutamine in inhibiting GAB with a Hill coefficient of ~1.3-1.4 and an independent manner in inhibiting KGA with a Hill coefficient of ~1.0-1.2 (Figure 1B & 1C). In conclusion, the mechanisms by which AV-1 inhibits GAB and KGA are different.

### Homologous modeling, docking studies, and binding site analysis

**Figure 2:**
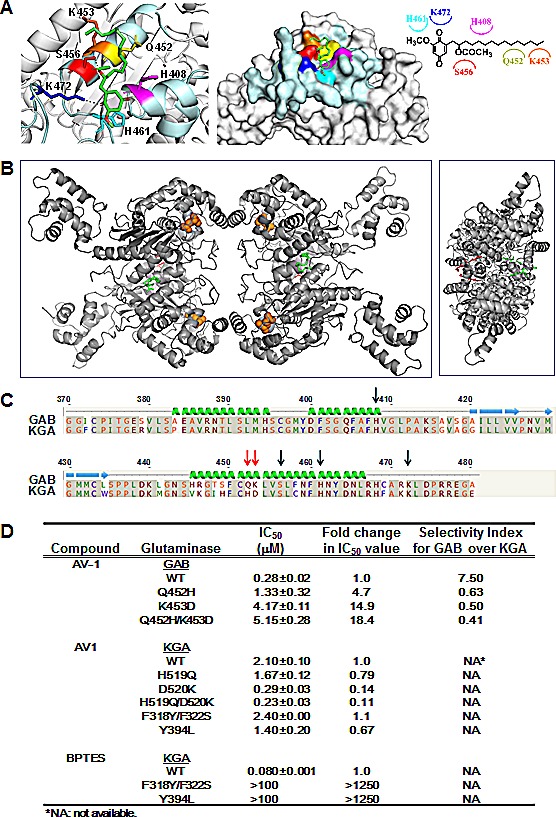
Docking studies and binding site analyses of AV-1 in GAB (A) Docking simulation of AV-1 binding site in GAB. Magenta: H408 histidine; Yellow: Q452 glutamine; Orange: K453 lysine; Red: S456 serine; Cyan: H461 histidine; Blue: K472 lysine; Dashed lines: hydrogen bond interactions. AV-1 chemical structure was shown with interacted residues of GAB. (B) Overview of the proposed association of AV-1 in GAB tetramer. Left panel presents a top view and right panel a side view. AV-1 is presented in red and green stick figure for clarity, and the glutamate molecules are shown as orange spheres. (C) Primary sequence alignment of human KGA and human GAB around the simulated AV-1 binding site. Key interactive residues are denoted with arrows. Mutation sites for selectivity of GAB over KGA are denoted with red arrows. (D) IC_50_ values and selectivity index of AV-1 & BPTES for GAB or KGA wild-type and mutant enzymes. Mutations at residues Q452 and K453 of GAB into corresponding residues of KGA created GAB mutants GAB_Q452H, GAB_K453D, and GAB_Q452H/K453D. Mutations at residues H519 and D520 of KGA into corresponding residues of GAB created KGA mutants KGA_H519Q, KGA_D520K, and KGA_ H519Q/D520K. Mutations at residues F318/F322 and Y394 of KGA into corresponding residues of GAB or bacterial glutaminase created KGA mutants KGA_F318Y/F322S and KGA_Y394L [3].

Based on the structure-activity relationships and inhibition modes analyzed above, we sought to determine the possible location of the AV-1-binding site on GAB [3]. Homologous modeling for GAB was conducted using the human GLS1 crystal structure 3UO9 and AutoDock Vina (http://vina.scripps.edu/). AutoDock Vina was further used to carry out a docking study for compound AV-1 over the entire surface of the homologous modeled GAB dimer or monoer. The simulated pocket for AV-1 binding from docking showed major hydrogen bond and van der Waals interactions between AV-1 with the GAB residues H408, Q452, K453, S456, H461 and H472 (Figure 2A). The binding pocket is located at the C-terminal end of the GAB monomer, close to the dimer interaction interface. However, no inter-monomeric interactions were found for AV-1 and therefore it is assumed that one AV-1 binds to one GAB monomer as shown in Figure 2B. Further sequence comparison of KGA and GAB at the AV-1 binding region indicate that the GAB residues Q452 and K453 were differentiated from those corresponding residues of KGA H519 and D520 (Figure 2C).

Mutagenesis studies to validate the interactions for selectivity were next carried out. When the residues Q452 and K453 in GAB were mutated into His or Asp to give the mutants Q452H, K453D or Q452H/K453D, the inhibitory effect significantly decreased: IC_50_ values of 0.28 ± 0.02 μM (wild type), 1.33 ± 0.32 μM (Q452H), 4.17 ± 0.11 μM (K453D), and 5.51 ± 0.28 μM (Q452H/K453D) were measured. These results demonstrate that the effects of the two GAB point mutations in decreasing AV-1 potency were additive. On the contrary, when the corresponding residues H519 and D520 in KGA were mutated into Gln or Lys to give the mutants H519Q, D520K or H519Q/D520K, the inhibitory effect increased: IC_50_ values of 2.1 ± 0.1 μM (wild type), 1.67 ± 0.12μM (H519Q), 0.29 ± 0.03 μM (D520K), and 0.23 ± 0.03 μM (H519Q/D520K) were measured. These results demonstrate that the effects of the two KGA point mutations reversely increased AV-1 potency.

Moreover, AV-1 comparably inhibited the wild type and the two KGA mutants corresponding to those of GAC (F318Y/F322S; Y394L) (Figure 2D) while these two KGA mutant enzymes were resistant to BPTES inhibition due to loss of key interactions demonstrated from co-crystallization data previously reported (Figure 2D) [3]. Therefore, we conclude that the interactions between AV-1 and GAB residues Q452 and K453 determine the selectivity for GAB over KGA.

### GLS2 inhibition via AV-1 compound or RNA silencing exhibits anti-cancer cell proliferation and anti- anchorage-independent colony formation

**Figure 3:**
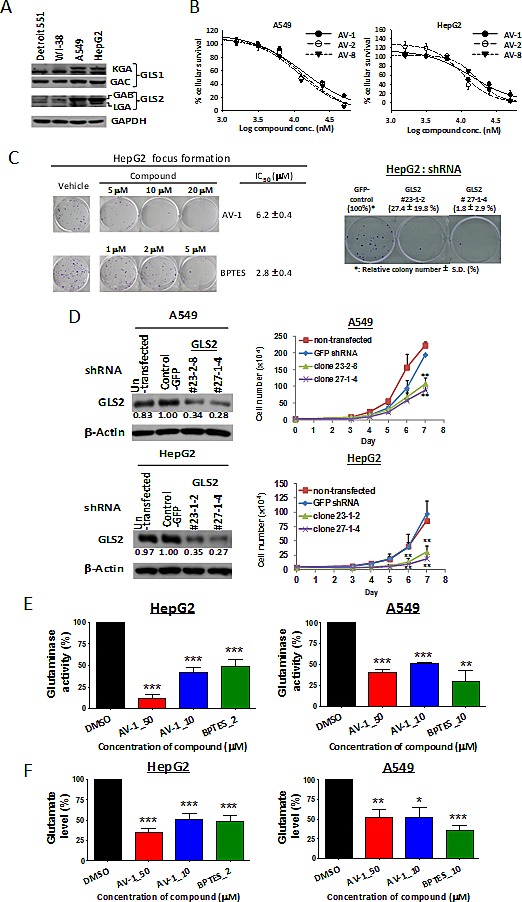
GLS2 inhibition via AV-1 compound or RNA silencing exhibits anti-cancer cell proliferation and anti-anchorage-independent colony formation (A) GLS1 and GLS2 protein expressions in carcinoma cells HepG2 and A549 as well as normal fibroblast cell lines Detroit 551 and WI-38. (B) Dose dependent growth inhibition of active alkyl benzoquinones against carcinoma cells HepG2 and A549. (C) The effect of GLS2 inhibition via AV-1 or GLS2 knockdown on the colony formation of HepG2 cells. (D) Knockdown GLS2 expression significantly inhibited the carcinoma HepG2 and A549 cells growth. Shown are western blot analysis for GLS2 expression levels and growth curves of un-transfected (parental) cells, shRNA-control cell (shRNA-control) and GLS2 shRNA knockdown cells. **P<0.005. (E) AV-1 treatments decreased intracellular glutaminase activity. (F) AV-1 treatments decreased intracellular glutamate levels. The cells lysates treated with AV-1 or BPTES were subject to glutaminase activity assay and glutamates levels (see experimental procedures). * P < 0.05, ** P < 0.01, *** P < 0.001. See also Figure S1 and Table S1.

GAB and KGA protein expression levels were found to be higher in human hepatoma HepG2 and lung carcinoma A549 cells than those in fibroblast cell lines Detroit 551 and WI-38, whilst GAC expression levels were not significantly changed (Figure 3A). LGA protein expression was low in each cell line but expressed slightly more in HepG2. KGA exhibited two major forms of protein, one of which was multi-phosphorylated as demonstrated by an addition of phosphatase experiment that affected the phosphorylation of KGA and changed the electrophoretic mobility of KGA protein in SDS gel (Figure 3A & Figure S1). The capacity of these compounds to inhibit growth of the cell lines A549, HepG2, MCF7 (human breast carcinoma), NCI-H460 (human lung carcinoma), and SF268 (human glioblastoma) (Figure 3B, Table S1, and previously described [27, 28]) were found to be consistent with their glutaminase inhibition (Table 1). Moreover, AV-1 inhibited colony formation of HepG2 cells, as did GLS1 inhibitor BPTES, a reference control (Figure 3C, left panel). After further knocking down GLS2 protein expression using a silencing RNA approach (see Materials and Methods), the A549 and HepG2 cell growth were decreased by ~45-81% (Figure 3D) and colony formation inhibited accordingly (Figure 3C, right panel). AV-1 treatment significantly decreased intracellular glutaminase activity and product glutamate levels (Figure 3E & 3F). Therefore, the alkyl benzoquinones inhibited carcinoma cell growth by inhibiting cellular glutaminase activity.

### AV-1 compound induces autophagy and activates AMPK

**Figure 4:**
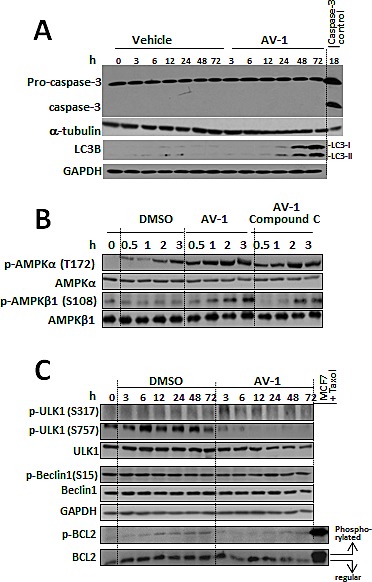
AV-1 induced cell autophagy through activated AMPK and ULK1 (A) Effects of AV-1 treatment on the induction of apoptosis and autophagy in HepG2 cells manifested by cleavage caspase 3 or LC3B. (B) The effects of AV-1 on AMPK activation. C. The effects of AV-1 on activation of ULK1 and BCL2 associated Beclin1. HepG2 cells were treated with vehicle (DMSO) or AV-1 for various times as indicated prior to cell lysis for western blot analysis with the antibodies indicated. The results shown are representative of three independent experiments.

Anti-cancer agents usually induce diversified forms of cell death including apoptosis and autophagy [29]. To examine whether AV-1 inhibited cell growth leads to cell death, the cell death-related apoptosis and autophagy pathways were examined in AV-1 treated HepG2 cells. Autophagic activity was monitored by tracking the conversion of LC3-I to LC3-II. A reliable marker of autophagosome LC3 is a mammalian homolog of yeast Atg8 [30]. It was found that LC3-II production was induced after the treatment of HepG2 cells with AV-1 for 24-72 h (Figure 4A), but the apoptosis marker-cleaved caspase 3 [31] was not detected (Figure 4A). Because glutaminase inhibition in cancer cells decreases cellular energy production and results in nutrient depletion [5] (Figures 3 B-F); and because autophagy is generally activated by conditions of nutrient deprivation [16], we concluded that induction of autophagy by AV-1 treatment was responsible for cell death (Figures 3B-E and 4A).

Autophagy is initiated and promoted by activation of AMPK_ULK1 axis signaling, but inhibited by mTORC1 [16]. Therefore, we examined the effects of AV-1 treatment on these signaling pathways in HepG2 carcinoma cells. Our investigation of the autophagy-related AMPK signaling pathway regulated by AV-1 showed: i) AMPK was phosphorylated for activation 0.5-3 h after AV-1 treatment (Fig 4B); ii) the moderate phosphorylation of ULK1 at Ser317 by AMPK [16] occurred at 3-12 h while the phosphorylation of ULK1 at Ser757 by mTOR [16] was significantly decreased up to 72 h after AV-1 treatment (Fig 4C); iii) The protein and phosphorylation (at Ser15) levels of autophagic Beclin1 were not significantly affected and the level of phosphorylated BCL2 decreased up to 72 h after AV-1 treatment (Figure 4C). These results indicate that the AMPK and ULK1 were activated while mTORC1 activity was suppressed in HepG2 cells treated with AV-1. Moreover, AV-1 induced autophagy is independent of Beclin1 and phosphorylated BCL2 signaling.

### AV-1 compound treatment results in mTORC1 inhibition through activated AMPK and inhibited P13K/Akt

**Figure 5:**
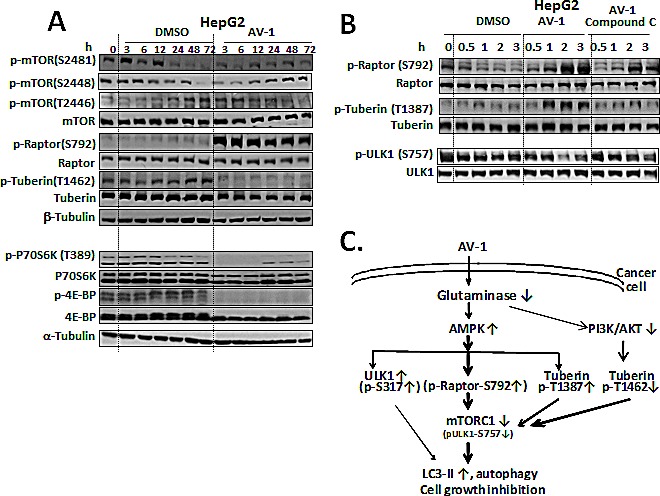
AV-1 inhibits mTORC1 through AMPK activation (A) Effects of AV-1 on mTORC1 inactivation. (B) AMPK inhibition reverses the AV-1 associated mTORC1 inhibition. HepG2 cells were treated with vehicle (DMSO), AV-1, or Compound C (an inhibitor of AMPK) for various times as indicated prior to cell lysis for western analysis with the antibodies indicated. (C) A scheme illustrating that AV-1 inhibits glutaminase, activates autophagic pathway through AMPK activation and mTORC1 inhibition. The results shown are representative of three independent experiments. See also Figure S2.

To investigate how mTORC1 activity is regulated by AV-1, phosphorylation of the mTORC1 components mTOR, Raptor, and Tuberin were separately examined (Fig. 5A). Firstly, phosphorylation at Ser2448 and auto-phosphorylation at Ser2481 of mTOR for activation via PI3K/AKT [32, 33] started to level off around 12 h, and then gradually increased until phosphorylation was complete at 72 h in AV-1 treated HepG2 cells. On the contrary, gradually decreasing levels of phosphorylation for these two sites occurred in the DMSO vehicle treatment.

Secondly, another nutrient sensing residue, Thr2446 of mTOR, which undergoes phosphorylation by AMPK to give inactivated mTOR under conditions of nutrient deprivation [34], underwent significant phosphorylation in the first 24 h, only to become dephosphorylated around 48-72 h in HepG2 cells treated with AV-1. On the contrary, HepG2 cells treated with DMSO vehicle did not show phosphorylation at Thr2446 of mTOR in the first 12 h, but a gradual increase in phosphorylation occurred from 24 h up to 72 h (Figure 5A). These results suggest that mTOR activity was inhibited for the first 12 h after AV-1 treatment, after which it became activated again.

Thirdly, residue Thr1462 of Tuberin (an AKT phosphorylation site to activate mTORC1) [35] was phosphorylated all over the 72 h-time course and even getting highly phosphorylated at 48-72 h period in the HepG2 cells treated with DMSO. However, AV-1 treatment disabled Tuberin phosphorylation at Thr1462 over the same time period (Figure 5A). These results suggested that AV-1 treatment likely inhibited PI3K/AKT activity and the resultant Tuberin (dephosphorylated at Thr1462) did contribute to inhibition of the mTORC1 activity.

Fourthly, a dramatic increase in phosphorylation of Raptor at Ser792 (a site for AMPK direct phosphorylation) for mTORC1 inactivation upon AV-1 treatment was observed compared to DMSO vehicle controls (Figure 5A upper panel).

Fifthly, mTORC1 inhibition was further manifested by the elimination of phosphorylation of p70S6K and 4E-BP, further downstream targets of mTORC1 by AV-1 treatment (Figure 5A lower panel).

Lastly, compound C (an AMPK inhibitor) attenuated the AV-1 induced phosphorylation of Raptor at Ser792 and Tuberin at Ser1387 (Figure 5B). In response to nutrient deprivation, Ser1387 of Tuberin gets phosphorylated by AMPK which also leads to mTORC1 inactivation [36]. On the contrary, compound C reversed the decreased phosphorylation of ULK1 Ser757 (Figure 5B and Figure S2).

Thus, we conclude that AV-1 inhibited intracellular glutaminase activity and resulted in decreased glutamate level in HepG2 cells, leading to autophagy by AMPK activation. AV-1 treatment activated AMPK which moderately phosphorylated ULK1 at Ser317 for autophagy initiation, and strongly phosphorylated Raptor at Ser792 and Tuberin at Ser1387 for mTORC1 inactivation for ultimate autophagy. Figure 5 C illustrated the effects of AV-1 in cell growth inhibition and induction of autophagy through AMPK mediated moderate ULK1 activation and profound mTORC1 inactivation.

## DISCUSSION

Herein, we report that natural product alkyl benzoquinones are novel inhibitors of the emerging cancer drug target glutaminase, a discovery made by screening a small collection of natural products. The chemical structures of the AV and AK compounds include a fatty acid moiety, reflecting the fact that fatty acids and fatty acyl-coenzyme A derivatives can modulate glutaminase activity [9]. Thus, the possibility arises that potent endogenous modulators may exist and play significant roles in regulating glutaminase activity under various physiological conditions.

GLS2 expression was reported to be significantly increased in 186 human colon carcinoma samples. GLS2 depletion in HT1299 lung cancer cells by siRNA increased the apoptosis cell population and, in combination with the inducers of ROS-dependent apoptosis doxorubicin or n-butyrate, further augmented the percentage of apoptotic cells [12]. These findings suggest a potential role of GLS2 in anti-cancer therapies. In our study, the relative inhibitory activities of the alkyl benzoquinones of glutaminase were generally consistent with their respective activities for anti-cancer cell growth (Table1, Table S1, and Figure 3B). Moreover, the GLS2 depletion in HepG2 cells also diminished cell growth (Figure 3D). Furthermore, AV-1 and GLS2 depletion significantly suppressed the colony formation of HepG2 (Figure 3C). Inhibition of glutaminase activity in cancer cells by alkyl benzoquinones results in a deficient nutrient supply (Figure 3E), which leads to i) an AMPK-mediated moderate ULK1 activation; ii) AMPK-mediated phosphorylations on Raptor Ser792 and Tuberin Thr1387 and a PI3K/AKT inhibition mediated dephosphorylation on Tuberin Thr1462, which results in significant mTORC1 inhibition; and ultimately iii) induction of autophagy (Figures 4 & 5). Therefore we conclude that GAB/GLS2 inhibition plays a significant role in the anti-cancer mechanisms for compound AV-1.

Glutaminase inhibitors are poorly explored, with few classes of potent allosteric inhibitors identified, e.g. BPTES and dibenzophenanthridine-968 compounds [2-6, 10]. Co-crystallization data showed that BPTES selectively inhibits GLS1 (KGA and GAC) via binding a differentiated gating loop close to the glutamine substrate binding site and exerting an allosteric effect to lock the tetramer into a nonproductive conformation. The GAC point mutants GAC F318Y/F322S and GAC Y394L were resistant to BPTES inhibition indicating that these interacting residues, Phe 322 and Tyr 394, are important for BPTES selectivity [3]. Molecular docking studies were conducted to locate potential dibenzophenanthridine-968 binding sites and site-directed mutagenesis performed to validate the important interacting residues. For example, two key residues Arg 539 and Phe 532 were mutated to construct R539L and F532L GAC mutants; dibenzophenanthridine-968 was found to be a greatly inferior inhibitor of GAC mutants than wild type. In order to understand more about the binding site of AV compounds, homologous modeling, docking and mutagenesis studies were used to propose an AV-1 binding site in GAB, and the roles of interacted residues in AV-1 selectivity for GAB over KGA. The analysis of structure-activity relationships reveals the importance of the two keto or hydroxyl group at positions 1 and 4 at the benzoquinone core and an acetate group at position 2' for AV-1 potency in inhibiting KGA and GAB. Docking studies found these side groups of AV-1 to interact with conserved residues Ser456 and Lys472 at GAB through hydrogen bonds (Figure 2A). In addition, replacement of the GAB residues Q452 and K453 with the corresponding KGA residues His519 and Asp520 affected glutaminase selectivity of AV-1 for GAB (GLS2) over KGA (GLS1) (Figure 2A & 2D), presumably because these residues interact with AV-1 through van der Waal forces. We constructed two KGA mutants corresponding to those of GAC (F318Y/F322S; Y394L) and tested their activities. As expected, BPTES was not a potent inhibitor of these two KGA mutants, whilst AV-1 inhibited both wild type KGA and the mutants to a similar degree (Figure 2D). This divergent differentiation of the isoforms of GAB and KGA presents the possible selective allosteric binding sites for GLS1 over GLS2 or GLS2 over GLS1. Moreover, these results clearly demonstrate that the proximal binding site of AV-1 was different from those of BPTES and dibenzophenanthridine-968.

The amino acid sequences of the glutaminase isoforms are highly homologous (81% identity) within their catalytic core regions (Figure S3, [37-39]). The rest of heterogeneous regions present potential differentiated allosteric pockets subjected to various modulations in enzymatic activity as evidence by inhibitors BPTES, dibenzophenanthridine-968, and AV-1. The accessibility of these allosteric pockets to small physiological molecules would allow for physiological modulations in the enzymatic activity. Thus, the possibility of identifying more allosteric pockets for modulating glutaminase isozymes merits further investigation.

## Materials and Methods

### Cell culture, reagents, and western blotting

The cancer cell lines including HepG2 and A549 were obtained from American Type Culture Collection (Rockville, MD). The cells were cultured in DMEM medium with 10% FBS (v/v) and penicillin (100 units/ml)/ streptomycin (100 mg/ml). Cultures were maintained in a humidified incubator at 37°C in 5% CO_2_/95% air. Dulbecco's Modified Eagle Medium (DMEM), fetal bovine serum (FBS), penicillin, streptomycin, and all other tissue culture reagents were obtained from GIBCO/BRL Life Technologies (Grand Island, NY). Antibodies to GAPDH, caspase 3, LC3B, p-ULK1(S317), p-ULK1(S757), Beclin1, p-BCL2(S70), p70S6K, p-p70S6K(T389), 4E-BP1, α-tubulin, 4E-BP, p-4E-BP(S65), p-eIF4B(S422), mTOR, p-mTOR(S2481), p-mTOR(S2448), p-Raptor(S792), Tuberin, p-Tuberin(T1462), p-Tuberin(T1387), p-AMPK(T172) and p-AMPK(S108) were from Cell Signaling Technology; antibody to p-Beclin1(S15) was from Abbiotec; antibodies to KGA and Raptor were from Abcam; antibodies to BCL2 were from Upstate Biotechnology; antibodies to α-tubulin were from Chemicon International; antibodies to ULK1, β-tublin and AMPK were from GeneTex; antibodies to GAB were from abnova and Gene Tex; antibodies to GAC were from ProteinTech Group; antibodies to p-mTOR(S2446) and AMPK were from Millipore; anti-mouse and anti-rabbit IgGs were obtained from PerkinElmer. Alkyl benzoquinones were purified from *A. virens* and *A. kusukuensis* [27, 28]. Compound C was obtained from Sigma–Aldrich (St. Louis, MO). Western blotting was performed as described [40].

### Preparation of BPTES

The synthesis of BPTES was modified from one previously published [41]. Step 1 Procedure: A mixture of thiosemicarbazide (1.82 g, 20 mmol), thiodipropionic acid (1.78 g, 10 mmol) and POCl_3_ (15 ml) was heated at 90 °C for 3 h. After obtaining a clear solution, the reaction mixture was cooled and poured in to 100 g of ice. The solid was filtered and the filtrate was adjusted to pH 14 using 5N NaOH. The resulting white solid was filtered, washed with water and dried to give bis(aminothioldiazole) (2.3 g, 80%).

Step 2 Procedure: NaH (60%, 660 mg, 16.5 mmol) was added to a solution of *bis*-(aminothioldiazole) (1.586 g, 5.5 mmol) in THF (200 mL) at 0°C. After being stirred for 5 min, phenylacetyl chloride (2.2 mL, 16.5 mmol) was added to the reaction mixture and the mixture was continuously stirred at room temperature for 16 h. Water was added to quench the reaction and the resulting mixture was extracted with ethyl acetate. The organic layers were washed with brine, dried over sodium sulfate, filtered, and concentrated *in vacuo* to furnish the crude BPTES. The solid was redissolved in DMSO and triturated with methanol to give BPTES as a white solid (518 mg, 18%) m.p 234-235 °C; ^1^H-NMR (300 MHz, (CD_3_)_2_SO): 2.92 (4H, t, *J*=7.2 Hz), 3.24 (4H, t, *J*=7.2 Hz), 7.31 (10H, m), 12.67 (2H, NH). Purity: 99.12% (RS-HPLC)

### Cloning, expression, and purification of glutaminases (KGA and GAB)

The cDNA clone MGC:33744 IMAGE:5263220 (Invitrogen Inc., USA) was used to amplify a KGA cDNA fragment of 1644 bp (residues Leu123-Leu669) by PCR with primers 5'-ATACGCGGATCCCTGGTGGCC TCAGGTGAAAA-3' and 5'–GTAAAGGAAAAAA GCGGCCGCTTACAACAATCCATCAAGAT-3' containing BamHI and NotI, respectively, for subsequent sub-cloning. The cDNA fragment of KGA digested with BamHI and NotI at each end was ligated into the pET28a(+) vector (Novagen) and expressed as a his-tag fusion. *Escherichia coli* strain BL21(DE3)pLysS was transformed with the resulting plasmid, pET28a(+)-KGA, and then cultured in Luria-Bertani broth medium in the presence of kanamycin. Once the cultures reached an absorbance at 600 nm of 0.5 to 0.6, they were induced with 0.5 mM isopropyl β-D-1-thiogalactopyranoside (IPTG) overnight. The cells were harvested by centrifugation at 10,000 rpm for 15 min at 4°C and resuspended in lysis buffer (5 mM Tris-HCl [pH 8.0], 50 mM NaH_2_PO_4_, 300 mM NaCl and 10 mM imidazole) for sonication. The filtered supernatant was applied to an affinity column, HisTrapTM HP 1 × 5 mL (GE Healthcare), and washed with lysis buffer containing 10 mM β-mercaptoethanol (β–ME), 5% glycerol, 0.1% Triton X-100 and 100-150 mM imidazole. His-tagged KGA was eluted with lysis buffer containing 10 mM β–ME, 5% glycerol, 0.1% Triton X-100 and 500 mM imidazole. The eluted protein was exchanged to 20 mM Tris-HCl (pH 8.0), 300 mM NaCl, 10% glycerol, and 2 mM dithiothreitol by using a PD-10 Desalting column (GE Healthcare). The concentration of the purified protein was determined by use of the Bio-Rad protein assay reagent with a standard curve plotted against bovine serum albumin.

Equivalent construct of GAB (Pro56-Val602) was amplified from the cDNA clone MGC: 195512 IMAGE: 100066375 (Invitrogen Inc., USA). The sequences of the primers were: 5'- GGAGGCGCGGATCCCCGC AGCACCAGGATCATG-3' (BamHI) and 5'- GCAAGGAAAAAAGCGGCCGCAGCG CTATACCATGCTTTCTAAGTTCTC-3' (Notl). The resultant GAB-expression vector, pET28a(+)-GAB, was transformed into *Escherichia coli* strain C41(DE3)pLysS (Yeastern Biotech Co., Taiwan). The expression protocol was followed as that protocol of KGA construct. The cells after induction were collected and resuspended in lysis buffer (50 mM Tris-HCl pH 8.5, 500 mM NaCl, 10% glycerol, and 10 mM β–ME) for sonication. The filtered supernatant was applied to a Ni^+^-charged affinity column (GE Healthcare), and washed with lysis buffer containing 50 mM imidazole. His-tagged GAB was eluted with lysis buffer containing 200 mM imidazole. The eluted GAB was exchanged to 20 mM Tris-HCl (pH 8.0), 300 mM NaCl, 50% glycerol, and 2 mM dithiothreitol by using a PD-10 Desalting column (GE Healthcare).

### Glutaminase activity assays and kinetic studies

Human glutaminase activity was measured using a two-step assay as described previously [42, 43]. A microtiter assay format was used to facilitate the collection of multiple data points. Typically, 10 μl of compound, which was dissolved in DMSO, was added to 80 μl of an initial assay mix (0.1 mM hKGA, 0.2 mM EDTA and 50 mM Tris/acetate, pH 8.6). Samples were incubated at 25°C for 10 min and then 10 μl of 200 mM glutamine were added to the reaction mix. After adding glutamine, the reactions were incubated at 37°C for 60 min and then quenched by the addition of 10 μl of 0.6 M HCl. Subsequently, 100 μl of a second reaction mixture (3.7 units of purified bovine liver glutamate dehydrogenase, 160 mM Tris/acetate [pH 9.4], 400 mM hydrazine, 5 mM ADP and 2 mM NAD^+^) was added to the stopped first reaction mixture and incubated for 30 min at 25°C. The absorbance at 340 nm was measured using a Wallac Victor2V plate reader (PerkinElmer), fitted with a 340 nm excitation filter. Sample absorbance was measured against a blank, containing no human glutaminase.

The kinetic constants K_m_ and V_max_ were determined by using Kaleida Graph software (Synergy Software) to calculate a non-linear least-squares fit for all the data points to the Michaelis–Menten equation. The Ki values for AV-1 were determined by using the Cheng-Prusoff equation; Ki=IC_50_/(1 + [S]/K_m_) where [S] is the concentration of the glutamine.

### *In vitro* carcinoma cell growth inhibition and colony formation assays

A549 lung carcinoma and HepG2 hepatocarcinoma cells were seeded at 4000 and 10000 cells/well respectively in 96-well plates, as previously described for cell growth inhibition assay [27, 44]. To assess anchorage-dependent colony formation effect, the cells (1000 cells/well) were seeded in a six-well plate for 20-24 h prior to treatments of vehicle DMSO or compounds as indicated. Culture medium containing vehicle DMSO or compounds as indicated was changed twice a week. After a 21-day treatment, the cell colonies were rinsed with PBS, fixed in 3:1 ratio of methanol and acetic acid, stained with 0.05% crystal violet.

### Determination of intracellular glutaminase activity and glutamate levels

Determination of intracellular glutaminase activity was modified from above described two-step glutaminase assay. Step one mixture of a 100 μl solution containing 20μg cell lysate, 0.2 mM EDTA, 50 mM Tris/acetate (pH 8.6), and 150 mM phosphate, was incubated at 37°C for 60 min and the reaction was stopped by 10 μl of 0.6 M HCl. Subsequently, 30 μl of the stopped reaction was subjected to the second reaction as described above for glutaminase assay. Glutamate levels were measured by using the Amplex Red Glutamine Acid/Glutamate oxidase assay kit (Invitrogen) according to manufacturer's instructions.

### Mutagenesis and docking

Site-directed mutagenesis was performed on the pET28a(+)-GAB or pET28a(+)-KGA plasmids using *PfuUltra* II Fusion HS DNA polymerase (Agilent Technologies) to create the GAB and KGA mutants. The deep view program (Version 3.7) of the Swiss-MODEL server was used to generate the three dimensional structure of homologous modeled GAB. Using the integrated sequence alignment tools and structural superimposition algorithms, the target sequence was mapped on the modeling templates. The sequence alignment was compared with crystal structure of the GAC (PDB code: 3UO9) and the resulting model of GAB was analyzed using the Swiss-PDB server. Threading and folding recognition of GAB model was developed by PHYRE2 web server. Docking studies were performed with AutoDock Vina. Autodock input files were prepared with MGLTools 1.5.6. Molecules were drawn in ChemBioOffice 2010, and energy minimized using the MM2 force field in Chemdraw 3D. Visualization was performed with PyMOL 1.3, and graphics were prepared in that software. SWISS MODEL: http://swissmodel.expasy.org/ and PHYRE2: http://www.sbg.bio.ic.ac.uk/phyre2/html/page.cgi?id=index.

### GLS2 gene silencing

The knock down of GLS2 was carried out using two distinct shRNA plasmids (clone ID: TRCN0000051323 and TRCN0000051327, Academia Sinica, Taiwan) directed against the two isoforms of GLS2 enzyme (GAB and LGA). The shRNA plasmid (TRCN0000231759, Academia Sinica, Taiwan) against of GFP was as a negative control. The shRNA plasmids were transfected in A549 cells, or HepG2 cells using FuGene 6^™^ (Roche). Stable cell clones were grown in the presence of 1 μg/mL puromycin. Established cell lines were further verified by anti-GAB western blot analysis to assess the cells growth.

### Funding

This work was funded by the Ministry of Economic Affairs, R.O.C. “101-EC-17-A-02-04-1099”, “102-EC-17-A-02-04-1099”, “103-EC-17-A-22-1099”, the National Science Council of Taiwan (NSC 102-2628-B-400-002-MY3), and the National Health Research Institutes, Taiwan, R.O.C.

## SUPPLEMENTARY MATERIAL TABLE AND FIGURES


