# Supplementary material for: Discovery of selective inhibitors of Glutaminase-2, which inhibit mTORC1, activate autophagy and inhibit proliferation in cancer cells
Source: Oncotarget. 2014 Jul 8;5(15):6087–101. doi: 10.18632/oncotarget.2173 (PMC4171615; doi:10.18632/oncotarget.2173)
Supplement: Supplementary file 1 [file oncotarget-05-6087-s001.pdf]

Discovery of selective inhibitors of Glutaminase-2, which inhibit mTORC1, activate autophagy and inhibit proliferation in cancer cells

Supplementary Material

Supplemental Table S1_related to Table 1. Human KGA and GAB inhibition of natural alkyl benzoquinones and their CC_{50} values and SD for cytotoxicity against carcinoma cells. see MATERILAS and METHODS for details.

	Cytotoxicity (cell line)	
	HepG2	A549
Compound ID	CC_{50} (μM)	CC_{50} (μM)
AV-1	11.6 ± 0.6	14.2 ± 2.8
AV-2	11.5 ± 0.9	12.8 ±1.5
AV-3	>50	>50
AV-4	>50	>50
AV-5	>50	>50
AV-6	>50	>50
AV-7	>50	>50
AV-8	18.8 ± 0.7	11.6 ± 0.4
AV-9	13.7 ± 2.2	13.0 ± 2.1
AV-10	21.2 ± 0.8	22.1 ± 1.6
AV-11	5.2 ± 0.3	5.0 ± 0.8
AV-12	>50	>50
AV-13	36.9 ± 2.0	35.5 ± 2.6
AV-14	>50	>50
AK-7	>100	>100
AK-12	84.6 ± 2.3	>100
BPTES	>50	>50

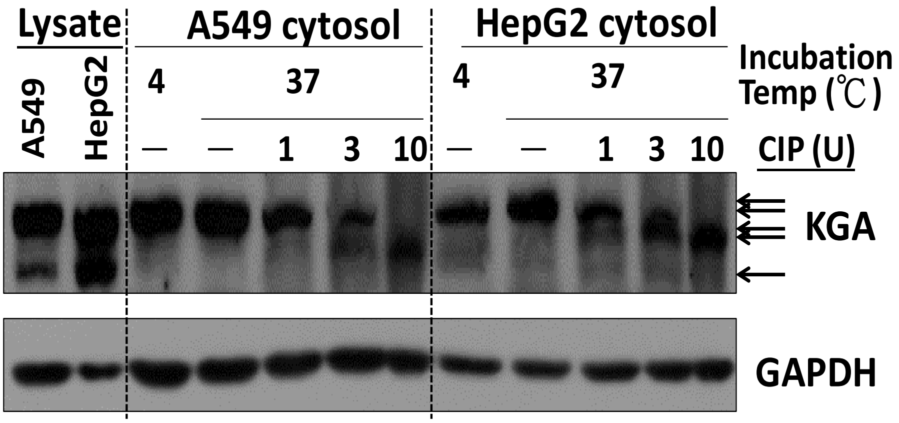

Supplemental Figure S1_related Figure 3A. A multi-phosphorylated KGA existing in the A549 and HepG2 cells. Human KGA exhibited two major protein bands in SDS-PAGE from the lysates of A549 and HepG2. The mobility of higher band was affected by the treatment of calf intestinal phosphatase (CIP, New England Biolabs) indicating a multi-phosphorylated KGA existing in the A549 and HepG2 cells. To prepare cytosol extracts, total cell lysates were resuspended in buffer containing 100 mM NaCl, 10 mM $MgCl_2$, 50 mM Tris-HCl, and 1 mM DTT, and kept at 4 ℃ for 10 min. The samples were centrifuged at 13,000 rpm for 10 min. The supernatants containing the solubilized cytosol proteins of A549 and HepG2 were incubated at 4 ℃ or 37 ℃ in the presence or absence of various units of CIP as indicated for 30 min prior to western analysis with antibodies for KGA (Abcam) or GAPDH (Cell Signaling) as a loading control. The results shown are representative of 3 independent experiments.

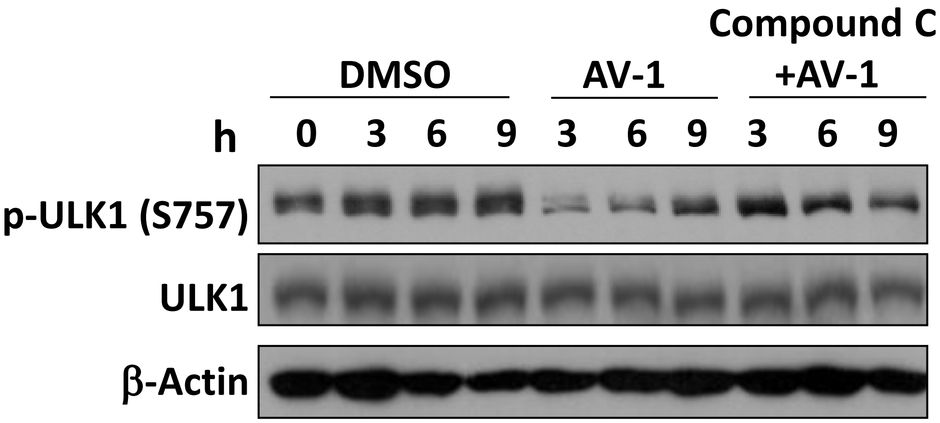

Supplemental Figure S2_related to Figure 5B. AMPK inhibition reversed the AV-1 associated autophagic inhibition in phosphorylation of ULK1 S757. HepG2 cells were treated with vehicle (DMSO), AV-1 (25 μM) or Compound C (20 μM) for 3 h, 6 h, and 9 h as indicated prior to cell lysis for western analysis with the antibody indicated. See Experimental Procedures for details. The results shown are representative of 3 independent experiments.

GLS1 and GLS2 alignment

```
                1                                                                                                 100
hGAB      (1)   ----------------------------------------MRSMKALQKALSRAGSHCGRGGWGHPSRSPLLGG------------------GVRHHLSE
hGAC      (1)   MMRLRGSGMLRDLLLRSPAGVSATLRRAQPLVTLCRRPRGGGRPAAGPAAAARLHPWWGGGGWPAEPLARGLSSSPSEILQELGKGSTHPQPGVSPPAAP
hKGA      (1)   MMRLRGSGMLRDLLLRSPAGVSATLRRAQPLVTLCRRPRGGGRPAAGPAAAARLHPWWGGGGWPAEPLARGLSSSPSEILQELGKGSTHPQPGVSPPAAP
hLGA      (1)   --------------------------------------------------------------------------------------------MLQKEEQV
                101                                                                                               200
hGAB     (43)   AAAQGRETPHSHQPQHQ---------DHDSSESGMLSRLGDLLFYTIAEGQERIPIHKFTTALKATGLQTSDPRLRDCMSEMHRVVQESSSGGLLDRDLF
hGAC    (101)   AAPGPKDGPGETDAFGNSEGKELVASGENKIKQGLLPSLEDLLFYTIAEGQEKIPVHKFITALKSTGLRTSDPRLKECMDMLRLTLQTTSDGVMLDKDLF
hKGA    (101)   AAPGPKDGPGETDAFGNSEGKELVASGENKIKQGLLPSLEDLLFYTIAEGQEKIPVHKFITALKSTGLRTSDPRLKECMDMLRLTLQTTSDGVMLDKDLF
hLGA      (9)   AVLRPHFPPHSLPP------------NSDSSESGMLSRLGDLLFYTIAEGQERIPIHKFTTALKATGLQTSDPRLRDCMSEMHRVVQESSSGGLLDRDLF
                201                                                                                               300
hGAB    (134)   RKCVSSNIVLLTQAFRKKFVIPDFEEFTGHVDRIFEDVKELTGGKVAAYIPQLAKSNPDLWGVSLCTVDGQRHSVGHTKIPFCLQSCVKPLTYAISISTL
hGAC    (201)   KKCVQSNIVLLTQAFRRKFVIPDFMSFTSHIDELYESAKKQSGGKVADYIPQLAKFSPDLWGVSVCTVDGQRHSTGDTKVPFCLQSCVKPLKYAIAVNDL
hKGA    (201)   KKCVQSNIVLLTQAFRRKFVIPDFMSFTSHIDELYESAKKQSGGKVADYIPQLAKFSPDLWGVSVCTVDGQRHSTGDTKVPFCLQSCVKPLKYAIAVNDL
hLGA     (97)   RKCVSSNIVLLTQAFRKKFVIPDFEEFTGHVDRIFEDVKELTGGKVAAYIPQLAKSNPDLWGVSLCTVDGQRHSVGHTKIPFCLQSCVKPLTYAISISTL
                301                                                                                               400
hGAB    (234)   GTDYVHKFVGKEPSGLRYNKLSLNEEGIPHNPMVNAGAIVVSSLIKMDCNKAEKFDFVLQYLNKMAGNEYMGFSNATFQSEKETGDRNYAIGYYLKEKKC
hGAC    (301)   GTEYVHRYVGKEPSGLRFNKLFLNEDDKPHNPMVNAGAIVVTSLIKQGVNNAEKFDYVMQFLNKMAGNEYVGFSNATFQSERESGDRNFAIGYYLKEKKC
hKGA    (301)   GTEYVHRYVGKEPSGLRFNKLFLNEDDKPHNPMVNAGAIVVTSLIKQGVNNAEKFDYVMQFLNKMAGNEYVGFSNATFQSERESGDRNFAIGYYLKEKKC
hLGA    (197)   GTDYVHKFVGKEPSGLRYNKLSLNEEGIPHNPMVNAGAIVVSSLIKMDCNKAEKFDFVLQYLNKMAGNEYMGFSNATFQSEKETGDRNYAIGYYLKEKKC
                401                                                                                               500
hGAB    (334)   FPKGVDMMAALDLYFQLCSVEVTCESGSVMAATLANGGICPITGESVLSAEAVRNTLSLMHSCGMYDFSGQFAFHVGLPAKSAVSGAILLVVPNVMGMMC
hGAC    (401)   FPEGTDMVGILDFYFQLCSIEVTCESASVMAATLANGGFCPITGERVLSPEAVRNTLSLMHSCGMYDFSGQFAFHVGLPAKSGVAGGILLVVPNVMGMMC
hKGA    (401)   FPEGTDMVGILDFYFQLCSIEVTCESASVMAATLANGGFCPITGERVLSPEAVRNTLSLMHSCGMYDFSGQFAFHVGLPAKSGVAGGILLVVPNVMGMMC
hLGA    (297)   FPKGVDMMAALDLYFQLCSVEVTCESGSVMAATLANGGICPITGESVLSAEAVRNTLSLMHSCGMYDFSGQFAFHVGLPAKSAVSGAILLVVPNVMGMMC
                501                                                                                               600
hGAB    (434)   LSPPLDKLGNSHRGTSFCQKLVSLFNFHNYDNLRHCARKLDPRREGAEIRNKTVVNLLFAAYSGDVSALRRFALSAMDMEQKDYDSRTALHVAAAEGHIE
hGAC    (501)   WSPPLDKMGNSVKGIHFCHDLVSLCNFHNYDNLRHFAKKLDPRREGGDQRHS--FGPLDYESLQQELALKETVWKKVSPESNEDISTTVVYRMESLGEKS
hKGA    (501)   WSPPLDKMGNSVKGIHFCHDLVSLCNFHNYDNLRHFAKKLDPRREGGDQRVKSVINLLFAAYTGDVSALRRFALSAMDMEQRDYDSRTALHVAAAEGHVE
hLGA    (397)   LSPPLDKLGNSHRGTSFCQKLVSLFNFHNYDNLRHCARKLDPRREGAEIRNKTVVNLLFAAYSGDVSALRRFALSAMDMEQKDYDSRTALHVAAAEGHIE
                601                                                                   670
hGAB    (534)   VVKFLIEACKVNPFAKDRWGNIPLDDAVQFNHLEVVKLLQDYQDSYTLSETQAEAAAEALSKENLESMV-
hGAC    (599)   ----------------------------------------------------------------------
hKGA    (601)   VVKFLLEACKVNPFPKDRWNNTPMDEALHFGHHDVFKILQEYQVQYTPQGDSDNGKENQTVHKNLDGLL-
hLGA    (497)   VVKFLIEACKVNPFAKDRWGNIPLDDAVQFNHLEVVKLLQDYQDSYTLSETQAEAAAEALSKENLESMV-
```

Supplemental Figure S3_related to Discussion for Figure 2. Amino acids sequence alignment for human GLS1(KGA, GAC) and GLS2 (GAB, LGA)(Holcomb, et al., 2000)(Gomez-Fabre, et al., 2000)(Elgadi, et al., 1999). The catalytic core regions are underlined and exhibit 81% homology as shown in red. The key residues involved in selectivity of BPTES (purple) or AV-1 (green) are denoted in arrows respectively.

Supplemental references:

Elgadi KM, Meguid RA, Qian M, Souba WW and Abcouwer SF. Cloning and analysis of unique human glutaminase isoforms generated by tissue-specific alternative splicing. Physiological genomics. 1999; 1(2):51-62.

Gomez-Fabre PM, Aledo JC, Del Castillo-Olivares A, Alonso FJ, Nunez De Castro I, Campos JA and Marquez J. Molecular cloning, sequencing and expression studies of the human breast cancer cell glutaminase. The Biochemical journal. 2000; 345 Pt 2:365-375.

Holcomb T, Taylor L, Trohkimoinen J and Curthoys NP. Isolation, characterization and expression of a human brain mitochondrial glutaminase cDNA. Brain research Molecular brain research. 2000; 76(1):56-63.
